# Spatial heterogeneity of nanomedicine investigated by multiscale imaging of the drug, the nanoparticle and the tumour environment

**DOI:** 10.7150/thno.38625

**Published:** 2020-01-01

**Authors:** Josanne Sophia de Maar, Alexandros Marios Sofias, Tiffany Porta Siegel, Rob J. Vreeken, Chrit Moonen, Clemens Bos, Roel Deckers

**Affiliations:** 1 Division of Imaging and Oncology, University Medical Center Utrecht, Utrecht University, the Netherlands.; 2 Department of Circulation and Medical Imaging, Faculty of Medicine and Health Sciences, Norwegian University of Science and Technology (NTNU), Trondheim, Norway.; 3 The Maastricht Multimodal Molecular Imaging Institute (M4I), Division of Imaging Mass Spectrometry, Maastricht University, Maastricht, the Netherlands.; 4 Janssen Research & Development, Beerse, Belgium.

**Keywords:** Drug distribution, Nanomedicine, Clinical Imaging, Optical imaging, Mass Spectrometry Imaging.

## Abstract

Genetic and phenotypic tumour heterogeneity is an important cause of therapy resistance. Moreover, non-uniform spatial drug distribution in cancer treatment may cause pseudo-resistance, meaning that a treatment is ineffective because the drug does not reach its target at sufficient concentrations. Together with tumour heterogeneity, non-uniform drug distribution causes “therapy heterogeneity”: a spatially heterogeneous treatment effect. Spatial heterogeneity in drug distribution occurs on all scales ranging from interpatient differences to intratumour differences on tissue or cellular scale. Nanomedicine aims to improve the balance between efficacy and safety of drugs by targeting drug-loaded nanoparticles specifically to tumours. Spatial heterogeneity in nanoparticle and payload distribution could be an important factor that limits their efficacy in patients. Therefore, imaging spatial nanoparticle distribution and imaging the tumour environment giving rise to this distribution could help understand (lack of) clinical success of nanomedicine. Imaging the nanoparticle, drug and tumour environment can lead to improvements of new nanotherapies, increase understanding of underlying mechanisms of heterogeneous distribution, facilitate patient selection for nanotherapies and help assess the effect of treatments that aim to reduce heterogeneity in nanoparticle distribution.

In this review, we discuss three groups of imaging modalities applied in nanomedicine research: non-invasive clinical imaging methods (nuclear imaging, MRI, CT, ultrasound), optical imaging and mass spectrometry imaging. Because each imaging modality provides information at a different scale and has its own strengths and weaknesses, choosing wisely and combining modalities will lead to a wealth of information that will help bring nanomedicine forward.

## Introduction

In 2015, 17.5 million people were diagnosed with cancer globally and its incidence is increasing. Although the prognosis of most cancer types has improved, still 8.7 million people died of cancer in that year 1. Rather than a single disease, 'cancer' comprises of a diverse collection of diseases. A high degree of heterogeneity in tumour genotype, phenotype and behaviour (including responsiveness to therapy) exists not only between tumour types, but also between tumours of the same histological type in different patients 2, between primary and metastatic tumours in the same patient, within a patient's tumour that is developing over time 3 and even within a single tumour at one moment 2, 4-10. The genetic and non-genetic causes of tumour heterogeneity have been reviewed in detail elsewhere. This heterogeneity is an important cause of therapy resistance 3, 11-14 and in some cancer types an association between the degree of intratumoural heterogeneity and a worse prognosis has been found 15-17. This stresses the importance of evaluating disease heterogeneity and the need for personalized treatment.

### Therapy heterogeneity

Therapy heterogeneity, a spatially heterogeneous treatment effect, is another important source of variability between patients and between tumours within an individual. Spatial heterogeneity in drug distribution contributes to therapy heterogeneity and can lead to pseudoresistance: the treatment does not have the desired effect, not because of cellular or genetic mechanisms of resistance, but because the drug simply does not reach all tumour cells at a high enough concentration 18-20. Moreover, unintended accumulation of drugs in healthy tissue may lead to increased toxicity 18. Furthermore, heterogeneity in spatial distribution of drugs can generate distinct microenvironments within the tumour, causing intra- and intertumour heterogeneity and, ultimately, influencing clinical outcome.

### Spatial heterogeneity of nanomedicine

Heterogeneous drug distribution occurs for drugs of all sizes. In this review we will focus on its impact in the field of nanomedicine. Nanomedicines are sub-micron size drug delivery systems, which are designed to improve the drug delivery to tumours while reducing systemic side effects 21. Several principles for drug targeting to tumours are described in literature 22: passive targeting (mainly relying on the enhanced permeability and retention (EPR) effect 23, 24), active targeting (using carriers decorated with tumour-specific targeting antibodies) or triggered release (drug release from nanocarriers in response to heat, ultrasound or light) 25. Regardless of the targeting method, nanomedicine has to overcome several physiological barriers before reaching the targeted tumour cells, which may very likely introduce therapy heterogeneity 26.

### Scales of heterogeneity

As reviewed by Garattini et al., heterogeneous drug distribution leading to therapy heterogeneity can occur on many scales 18. On each scale different factors influence the distribution of the nanoparticle and drug.

On patient scale, the inter-patient variability of nanomedicine pharmacokinetics (PK) is influenced by many factors such as age, gender, body composition, prior treatments, and drug-drug interactions 27. For a number of liposomal nanoparticles, it has been shown that the PK variability of nanoparticles is greater than that of the corresponding small molecule drugs 28. Clearance of most nanoparticles occurs mainly via the mononuclear phagocyte system (MPS, also known as the reticuloendothelial system) through uptake by circulating and tissue-homing phagocytic cells, primarily in the liver and spleen 29. One explanation for interpatient differences is that MPS function is affected by age, gender and inflammation 30. Comorbidity affecting renal function or hepatic function could in turn diminish renal clearance or hepatobiliary excretion of certain nanoparticles. On the other hand, the presence of tumours in the liver increased the clearance of a liposomal campthothecin analogue 31. Furthermore, due to the accelerated blood clearance (ABC) phenomenon, a second dosage of polyethylene glycol (PEG)ylated nanoparticles is cleared more rapidly, leading to additional inter and intra patient differences 32.

Also on the organ and tumour scale, many factors can contribute to heterogeneity in nanoparticle and drug distribution between different tumours in an individual patient. Accumulation of nanoparticles is tumour type and organ dependent 33. Tumour locations with limited perfusion or specific barriers such as the blood brain barrier 34 or blood retina barrier 35 can hinder nanoparticle and drug accumulation. A large variation in EPR effect exists between tumour types, sizes and locations, most likely related to variability in tumour blood vessel architecture and function 26, 36. Likewise, preclinical small animal tumour models generally overestimate the EPR effect compared to tumours in patients, which complicates clinical translation of nanomedicine 37. Moreover, differences in composition of the tumour microenvironment (including immune cell infiltration, pericyte coverage of the endothelium, density of the extracellular matrix, hypoxia and interstitial fluid pressure (IFP) can lead to intertumour heterogeneity in nanoparticle accumulation and treatment effect 3, 37-40.

Finally, on tissue and cellular scale, there are numerous causes for heterogeneity in drug and nanoparticle distribution within a single tumour. These intratumour differences relate among other things to endothelial cell gaps across the vessel wall, perfusion, extracellular matrix composition and immune cell presence (e.g. tumour associated macrophages, TAM) 3, 19, 38. Variable endothelial gaps (ranging from one to hundreds of nanometers) result in non-uniform extravasation of nanoparticles into the tumour 41. Heterogeneity in tumour perfusion will cause non-uniform transport of nanoparticles and nutrients to different parts of the tumour and introduce local variance in oxygenation and tumour pH 19, 38.

### Role of imaging to evaluate spatial heterogeneity of nanomedicine

Some nanomedicine formulations are currently used in the clinic 42, 43, but overall the success of nanomedicine has been modest 44-46. Spatial heterogeneity in distribution of nanoparticles could be an important factor limiting the efficacy in patients and therefore the acceptance of nanotherapy in the clinic. Better understanding of the extent and impact of therapy heterogeneity from cellular to patient scale may increase the success of nanomedicine in clinical practice.

Many imaging methods are available to visualize at a variety of scales the three main factors (i.e. nanoparticle distribution, drug distribution and tumour environment) that influence spatial therapy heterogeneity and thus efficacy. These methods can be used to:

1. Evaluate the effect of the therapy in a preclinical setting, to facilitate the development of new treatments.

2. Improve understanding of the underlying mechanisms that lead to heterogeneous distribution of nanoparticles and drugs.

3. Select patients and predict their treatment response for a personalized treatment plan: which patient likely benefits from a certain therapy and in which patient is adaptation of therapy necessary?

4. Evaluate the effect of methods that aim to address therapy heterogeneity, such as modulating the tumour microenvironment, hyperthermia or sonopermeation 19, 47, 48.

### Scope

Over the last years, several excellent review papers on imaging in nanomedicine were published focusing on imaging the biodistribution of nanoparticles 49, imaging labelled nanoparticles 50-52, the role of imaging to evaluate treatments that alter nanoparticle delivery 47, imaging nanoparticles as companion diagnostics 53, 54, or clinical applications of imaging in the field of nanomedicine 55-57. In this review we will provide a non-exhaustive overview of imaging methods used in the field of nanomedicine, which visualize spatial distribution of nanoparticles or drugs or factors contributing to heterogeneity on different scales, i.e. patient, organ/tumour and tissue/cellular scale. For each method, we will highlight the three main aspects that can be imaged: the drug, the nanoparticle and the tumour (micro-) environment. We will focus on three groups of imaging modalities. A summary of the modalities and their strengths and limitations is presented in Table 1. First we will discuss non-invasive clinical imaging methods, because of their direct usability in clinical translation of nanomedicine. Subsequently, we will elaborate on the most commonly used preclinical modality optical imaging as it is the most frequently employed imaging modality to investigate the interplay between drug, nanoparticle and environment. Optical imaging can be a non-invasive technique in the preclinical setting, while it is invasive clinically. Finally we will discuss mass spectrometry imaging (MSI), an invasive but promising and versatile label-free method for monitoring drug distribution and effect. Our goal is to show how imaging can provide information on all aspect that influence nanotherapy and in this way will help clinical and preclinical researchers to improve the effectiveness of nanotherapies and translate their use to cancer patients.

### Nuclear imaging: scintigraphy/SPECT/PET

Nuclear imaging techniques, namely scintigraphy, Single-Photon Emission Computed Tomography (SPECT) and Positron Emission Tomography (PET) provide highly sensitive quantitative information about the distribution of an administered radiopharmaceutical. They are often combined with CT (SPECT/CT or PET/CT) to add anatomical information and perform attenuation correction. Since the spatial resolution is lower than that of MRI, CT and US imaging, clinical PET and SPECT are mainly informative on patient and organ scale. In the preclinical setting, high-resolution PET and SPECT techniques demonstrate expansion to the tissue scale 58, which is nicely represented by the study of Wang et al. showing the heterogeneous spatial distribution of radiolabelled multi-walled carbon nanotubes in mouse brains with high-resolution SPECT 59.

Currently, the metabolic activity measured with ^18^F-fluorodeoxyglucose (FDG-) PET/CT is widely used in the clinic for diagnosis and monitoring therapy response. Traditionally several parameters are analysed for diagnosis and prognosis, including maximum standardized uptake values (SUVmax), peak standardized uptake values (SUVpeak), metabolic tumour volume (MTV), and total lesion glycolysis (TLG). Recently, intratumoural heterogeneity of baseline ^18^F-FDG uptake measured by PET texture analysis has been introduced as new predictive and prognostic factor for neoadjuvant chemotherapy 60-63.

### Imaging the nanoparticle

Nuclear imaging has already gained widespread acceptance in the management of cancer using standard chemotherapeutic agents, but may also play an important role in the advancement of nanomedicine towards clinical practice. Historically, nuclear imaging is used to image the distribution of radiolabelled nanoparticles on the patient and organ/tumour scale 47, 49-51, 56, 57. Already in 1984, Lopez et al. used scintigraphy to track the distribution of Tc-99-m labelled liposomes in cancer patients on organ scale 64. More recently, studies have used nuclear imaging of radiolabelled nanoparticles as a companion diagnostic to predict treatment response to drug containing nanoparticles or for directly visualizing the distribution of labelled therapeutic nanoparticles 65-69. The latter approach is nicely illustrated by a clinical study in nineteen HER2-positive metastatic breast cancer patients 69. HER2-targeted PEG-liposomes containing doxorubicin were administered together with a ^64^Cu-labelled tracer dose of the same liposomes and their distribution was imaged by PET/CT. This study was able to demonstrate and quantify the EPR effect in patients and found that tumour liposome concentrations were similar to those found preclinically. Heterogeneity in nanoparticle distribution on the patient and tumour scale was observed and an association was found between the amount of ^64^Cu-labelled liposome uptake in the tumour and the overall tumour response and progression free survival 69. Recently, Miedema et al. used PET/CT to track ^89^Zr-labelled docetaxel nanoparticles in five patients with various tumour types and observed uptake of the nanoparticles in 35% of the tumours, which they attributed to the EPR effect. Heterogeneous patterns of accumulation were seen on patient and organ scale 70.

### Imaging the tumour environment

Furthermore, clinical studies with different tracers are being conducted to extend the use of nuclear imaging to gaining insight into the different aspects of tumour physiology, such as proliferation (e.g. ^18^F-fluorothymidine (FLT-PET) 71-73), hypoxia (e.g. ^18^F-fluoromisonidazole (F-MISO)-PET 55, 74, 75) and angiogenesis (e.g. with radiolabelled arginine-glycine-aspartic acid (RGD) peptide tracers 76 or vascular endothelial growth factor expression 76, 77).

Other tracers visualize expression of receptors that can be targeted by drugs (e.g. ^18^F-fluoroestradiol (FES-) PET 78, 79, human epidermal growth factor receptor 2 (HER2-) PET or SPECT 80), demonstrate suitability for targeted therapy (e.g. response prediction to tyrosine kinase inhibitors in non-small cell lung cancer 76, 81), or image tumour specific markers (e.g. prostate specific membrane antigen (PSMA-) PET 82, 83 and radiolabelled somatostatin analogues for neuroendocrine tumours 84, 85).

**Figure 1:**
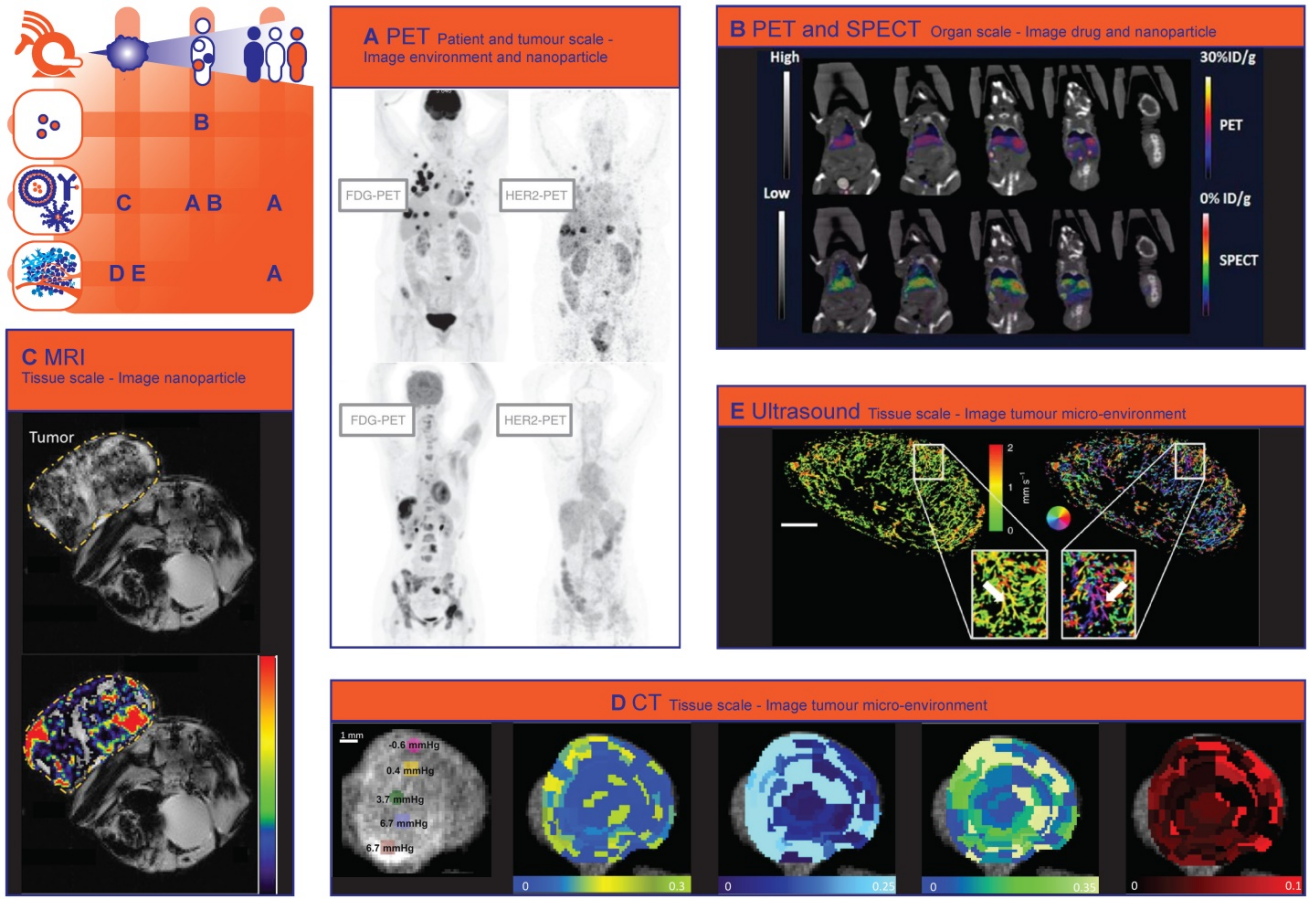
** Non-invasive clinical methods to image spatial heterogeneity of nanomedicine. (A)** Patterns of HER2-PET/CT confronted with FDG-PET/CT, Maximum intensity projection. Lesion uptake was considered pertinent when visually higher than blood pool. **Top**: dominant part of tumour load showed tracer uptake. Lung, liver and bone involvement seen of FDG-PET: not all lung lesions are seen on HER2-PET. **Bottom**: entire tumour load lacked tracer uptake. Liver and bone involvement seen on FDG-PET are not seen on HER2PET.* (Adapted with permission from 86, copyright 2016 Oxford University Press on behalf of the European Society for Medical Oncology). **(B)** In vivo* computed tomography (CT), positron emission tomography (PET)/CT, and SPECT/CT images of a nude mouse injected with 14 MBq of [^18^F]-FCP encapsulated [^111^In]-Liposome through tail vein injection 1 h post-administration. Coronal images. Both PET/CT and SPECT/CT images show the uptake of [^18^F]-FCP encapsulated in [^111^In]-Liposome in the liver and spleen. Both images correspond to each other in the uptake profile, demonstrating the feasibility of dual-tracer imaging from a single nano-construct. (*Adapted with permission from 89, copyright 2017 MDPI). **(C)***MR T2* images of CL1-5-F4/NF-κB-*luc2*-xenograft-bearing mice treated with erlotinib-conjugated iron oxide nanoparticles. Voxelwise estimates of the intratumoural iron concentration derived from changes in the ΔR2* signal (*P* < 0.0001), which correlates to the amount of intratumoural erlotinib content. **Top**: T2* weighted MR image. **Bottom**: T2*-weighted MR image with color-coded overlay of voxelwise estimates of intratumoural iron concentration* (Adapted with permission from 105, copyright 2018 Elsevier). **(D)*** A panel of images showing point-based measurements of IFP overlaid on the intratumoural distribution of CT-liposomes in an orthotopic tumour. Images from **left to right** represent: interstitial Fluid Pressure (IFP); permeability; perfusion; interstitial volume fraction; plasma volume fraction. The coloured circles and corresponding numbers represent the region of interest (ROI) locations, ROI size used for point-based analysis, and measured IFP. Predominantly peripheral CT-liposome enhancement was observed, with some heterogeneous accumulation within the central tumour region. Metrics of perfusion were spatially heterogeneous, but tended to increase towards the tumour periphery. *(Adapted with permission from 118, copyright 2015 Elsevier). **(E)***Motion model ultrasound localization microscopy (mULM). Super-resolution ultrasound images of an A431 tumour provide detailed information on the microvascular architecture including insights into vascular connectivity and the number of vascular branching points (see arrows in magnifications). Functional information such as MB velocities (**left** image) and MB flow directions (**right** image; color-coding illustrating the direction of flow according to the coloured circle) can be determined for each individual vessel and evaluated together with the morphological characteristics. Scale bar = 1 mm. *(Adapted with permission from 142, copyright 2018 Nature Research).*

Nuclear imaging methods create the possibility to combine imaging of nanoparticle distribution with imaging of characteristics of the tumour environment. A good example of this is the ZEPHIR trial 86, which was able to characterize tumour and therapy heterogeneity on the patient and tumour scale, based on pre-treatment HER2-PET/CT with ^89^Zr-trastuzumab and FDG-PET/CT after just one treatment cycle. These two types of PET imaging where used to characterize distribution of the nanoparticle (trastuzumab in T-DM1) and the tumour environment (FDG uptake). In metastatic breast cancer patients, who all had HER2 positive disease on biopsy, the combination of early metabolic response on FDG-PET (significant reduction in FDG uptake in >50% of the tumour load) and positive ^89^Zr-trastuzumab uptake (>50% of the tumour load) on the HER2-PET could predict treatment response to the antibody-drug conjugate (ADC) trastuzumab-emtansine (T-DM1) (Fig. 1A) 86. A preclinical immunohistochemistry study has shown that trastuzumab distribution is also very heterogeneous on the tissue scale and that a large amount of HER2 receptors are never reached by trastuzumab 87. The ZEPHIR study demonstrates that the inherent problems of characterising the tumour environment by tissue biopsies (i.e. sampling error and overlooking intra- and intertumour heterogeneity) can be circumvented through the use of nuclear imaging.

### Imaging the drug

Depending on the nanoparticles' release characteristics, imaging nanoparticle distribution might not necessarily reflect the distribution of encapsulated drugs and thus provide an incomplete view of drug distribution. To avoid the shortcomings of imaging the nanoparticle, the chemotherapeutic drug camptothecin and a photosensitizing agent were conjugated and labelled with ^64^Cu. In this way, the distribution of the drug conjugate could be imaged even after release from the polymeric nanoparticle. This study showed that a higher amount of the ^64^Cu-labelled photosensitizing agent was delivered to the tumour when it was conjugated to camptothecin. Accordingly, nanoparticles containing the combination more efficiently inhibited tumour growth than nanoparticles containing either the photosensitizer or camptothecin 88. Attempts have been made to distinguish the distribution of nanoparticles from the distribution of drugs with nuclear imaging. For example, Lamichhane et al. combined PET and SPECT to image [111In]-Liposomes and the encapsulated [^18^F]-Fluorinated Carboplatin separately on organ scale. A similar distribution was found for both drug and carrier, with the highest accumulation in the spleen and liver. (Fig. 1B) 89.

### Magnetic resonance imaging

Because of the lack of radiation, its high spatial resolution and excellent soft tissue contrast**.** Magnetic resonance imaging (MRI) is widely used in daily clinical practice for tumour diagnosis, characterisation and response evaluation. MRI is increasingly being investigated in image-guided therapy such as MR-guided radiotherapy (MR-LINAC) 90 and MR-guided High Intensity Focused Ultrasound (MR-HIFU) 91, 92. Several MRI techniques have been used in nanomedicine research, to characterize the tumour environment and to image the distribution of nanoparticles or (model) drugs on organ/tumour and tissue scale 47, 49, 50, 55, 56, 93.

### Imaging the tumour environment

Tumour vascular development and density, as well as perfusion and hypoxia, are key regulators of nanoparticle distribution and nanotherapy effect. Dynamic Contrast Enhanced (DCE-) MRI has been used in clinical trials to evaluate the effect of antivascular treatment on perfusion and vascular permeability 94, 95. Preclinically, Baker et al. used MRI and histopathology to evaluate factors of the tumour environment that contribute to therapy heterogeneity on tissue level. Distribution of trastuzumab was very heterogeneous. However, area's with little trastuzumab did not correspond with areas that were poorly vascularized 96. More specifically related to nanomedicine, Activin receptor-like kinase 5 (ALK5) inhibition with A-83-01 was shown to increase accumulation of liposomal Gadolinium (Gd) diethylenetriaminepentaacetic acid (DTPA) on dynamic MRI 97. Restricted diffusion on diffusion weighted imaging (DWI) correlates with the cell density of a tumour 98, 99, while Blood Oxygenation Level Dependent (BOLD-) and Tissue Oxygenation Level-Dependent (TOLD-) MRI quantify tumour oxygenation 100, 101. Hypoxic regions inherently have impaired transport of molecules and in addition hypoxia alters key cellular process such as energy metabolism and cellular receptor uptake and signalling, that can affect both intracellular uptake and efflux of nanomedicine 102.

### Imaging the nanoparticle

Iron-oxide nanoparticles have been approved as MRI contrast agent for clinical use. However, they can also function as companion diagnostic or as (imageable) drug delivery systems 103. For example, Ramanathan et al. conducted a clinical pilot study where they used feromoxytol (FMX) iron nanoparticles (also known as superparamagnetic iron oxide particles, SPION) to predict the deposition of nanoliposomal irinotecan. They showed a correlation between FMX-MRI and tumour response 104. This companion diagnostic approach could lead to improved patient selection and personalized treatment. Alternatively, tracking the distribution of therapeutic nanoparticles could help with response prediction and early adaptation of a treatment plan. For example Hsu et al. could track the uptake of iron oxide nanoparticles conjugated to tyrosine kinase inhibitor erlotinib at tissue scale, and observed that the particle induced tumour inhibition in non-small cell lung cancer-bearing mice (Fig. 1C) 105. Also other MRI contrast agents such as manganese and gadolinium have been incorporated in nanoparticles to create paramagnetic nanoparticles that can be imaged with MRI 106, 107. For instance, Nitta et al. used Gd-dendron modified liposomes to evaluate intratumoural microvasculature with MRI and found a clear difference in vessel architecture between two tumour models. In addition, increased leakage of the liposomes into the tumour tissue was observed after anti-angiogenic sunitinib treatment 108.

### Imaging model drugs

Moreover, MRI contrast agents have been encapsulated in nanoparticles as model drugs, to visualize and quantify drug release triggered by temperature, pH or ultrasound sensitive nanoparticles using MRI 93, 109. Onuki et al. combined two MRI contrast agents to visualize the nanoparticle distribution as well as content release in mice on tissue scale. The mice were treated with poly(D,L-lactide-*co*-glycolide) nano/microspheres, encapsulated with gadolinium-DTPA, SPIONs and the chemotherapeutic drugs 5-fluorouracil. *In vivo*, release of gadolinium-DTPA was seen from 30 minutes after intravenous injection, in the same tumour regions where most of the nanospheres had accumulated 110. Using MR contrast agents as a model drug is a convenient way to visualize *in vivo* drug release and spatial distribution at tumour and tissue scale. However, these MR contrast agents may influence the stability of nanoparticle 111 and interact with the co-loaded drug. Furthermore, the tissue distribution of the MR contrast agent and the co-loaded drug may not correspond due to different physicochemical properties of both molecules.

### Computed tomography

Computed tomography (CT) imaging is very commonly used in the clinic for diagnostic purposes and response evaluation after treatment. More recently, it was shown that CT could derive tumour transport properties in patients with pancreatic cancer that correlated with gemcitabine incorporation, pathological response, and oncologic outcome 112. Yoon et al. showed that CT texture features, as a non-invasive imaging biomarker for the identification of intratumoural heterogeneity, correlated with survival rate in gastric cancer 63.

### Imaging the tumour environment

The added value of CT imaging for nanomedicine through identifying tumour transport properties was already shown in the preclinical setting. Dynamic Contrast Enhanced (DCE) CT has been used in several studies to measure the intratumoural perfusion, permeability and the accumulation of CT contrast agent-containing nanoparticles in mice 49, 113-116. Since intratumoural perfusion is associated with liposome accumulation, DCE-CT could be useful to select patients more likely to respond to treatment with liposomal drugs 117. Correlations were found between distribution of interstitial fluid pressure, tumour perfusion and the intratumoural accumulation of iohexol-containing liposomes imaged with CT on tissue scale (Fig. 1D) 118. Spectral CT is another promising technique to image therapy heterogeneity on tissue scale, since it can provide high-resolution imaging and quantification of various components of the tumour microenvironment by taking advantage of differences in their energy-dependent attenuation 119. Spectral CT has already been utilized to monitor vascular and tumour response to vascular endothelial growth factor (VEGF-) inhibitors in rabbits 120 and to assess angiogenesis clinically 121. Related to nanomedicine, both tumour vasculature and tumour retention of liposomes has been imaged simultaneously with spectral CT, by administering iodine and Gd liposomes at different intervals before CT imaging 116.

### Imaging the nanoparticle

In addition to imaging a contrast agent encapsulated in a nanoparticle, CT can also be utilized to image metallic nanoparticles with a high attenuation of x-rays 49, 122. For example Mao et al. used CT to image the distribution of gold nanoparticle clusters containing doxorubicin on tissue scale and found that the nanoparticles accumulated mostly in the periphery of the tumour 123. Because vessels in the tumour periphery are actually on average less permeable than in the tumour core, these results suggest that EPR is not the only factor in play. Extravasation into the core might, among other factors, be hampered by tumour perfusion and the interstitial tumour matrix 26. In another study, CT imaging showed that hollow bismuth subcarbonate nanotubes, assembled from ultrasmall nanoclusters and loaded with doxorubicin for chemoradiotherapy, had an increased circulation time and exhibited a stronger EPR effect in mice compared to non-assembled ultrasmall nanoclusters 124.

### Ultrasound imaging

Ultrasound (US) is a low-cost, radiation-free and patient-friendly clinical imaging method that is mostly used for tumour diagnosis and image-guided biopsies. The introduction of contrast-enhanced ultrasound (CEUS), i.e. using microbubbles as ultrasonographic contrast agents, has extended the application of ultrasound in many fields due to improved image quality and new information that cannot be obtained with standard US 125, 126.

### Imaging the tumour environment

CEUS can provide anatomical as well as functional information about the vasculature of the tumour (micro-) environment 127-131. As an example, CEUS with poly (butyl cyanoacrylate)-based microbubbles has been used to image the degree of vascularisation on tumour scale in mice, which was correlated with the degree of EPR-mediated accumulation of a polymeric drug carrier 132. Moreover, Rojas et al. used targeted sub-micron phase-change contrast agents (liquid perfluorocarbon droplets, which contrary to microbubbles can also provide extravascular contrast) to image angiogenic vessels and perfusion in rats 133.

Tumour environment modelling treatments to improve nanoparticle delivery have been investigated using CEUS. Changes in tumour physiology (i.e. vessel fraction and blood flow) measured by US imaging after collagenase treatment corresponded with changes in IFP, therefore US imaging can be used as an earlier marker of tumour response 134. Fibrinolytic therapy decompressed blood vessels and improved tumour perfusion was observed with CEUS. Probably related to these physiological changes, the anticancer efficacy of nanoparticle-encapsulated paclitaxel and the penetration of liposomal doxorubicin improved by fibrinolytic therapy 135. IFP can also be measured directly by US elastography 136.

Recent advances in US imaging such as ultrafast ultrasound and super-resolution techniques provide also information on microvascular properties 137. Ultrafast Doppler imaging is capable of visualizing the heterogeneous tumour vasculature over time in 3-D with high sensitivity and spatial resolution (80 μm) 138, 139. Super-resolution ultrasound imaging technology allows vascular imaging at even higher spatial resolution (~10 μm) 140. These techniques have already been used for detailed visualization of tumour microvascular morphology 141, characterization of tumour perfusion on tissue scale (Fig. 1E) 142 and monitoring of early tumour response to an angiogenesis inhibiting drug 143 and will soon be of great value for prediction of nanotherapy heterogeneity and response.

### Imaging the nanoparticle

In addition to imaging the tumour environment, the distribution of nanoparticles can be imaged by US, using echogenic nanoparticles 144 (e.g. nanobubbles 145, 146, echogenic liposomes 147, polymeric gas-containing nanoparticles 148-150 or combining ultrasound contrast agents with nanoparticles through simultaneous administration or the use of nanoparticle-coated microbubbles 151-153. Besides the benefits associated with imaging of nanoparticle distribution, ultrasound and microbubbles can improve the therapeutic effect of a drug or nanoparticle through a number of mechanisms, summarized as 'sonopermeation' 154.

### Imaging the drug

Although drugs cannot be imaged directly with ultrasound, drug distribution can be visualized. To achieve this, Ektate et al. developed low-temperature sensitive echogenic liposomes, loaded with doxorubicin and perfluoropentane. Tumour hyperthermia led to increased US contrast in mice, which was correlated with increased doxorubicin delivery 147. Min et al. used a different approach and administered doxorubicin-loaded calcium carbonate polymeric nanoparticles to tumour-bearing mice. In an acidic environment, such as a tumour, the nanoparticles released their doxorubicin load and simultaneously produced carbon dioxide nanobubbles through hydrolysis, which made ultrasound imaging of release at tumour scale possible 155.

### Optical imaging of nanotherapy heterogeneity

Optical imaging modalities are used to collect a variety of information on various spatial and temporal scales 156: from organism to molecule and from static snapshots to real-time continuous dynamic visualization 157. These techniques are used in preclinical set-ups to provide macroscopic information at organism and organ scales (bioluminescence imaging (BLI), fluorescence imaging (FLI)) 158 or combined in tomographic set-ups to provide three-dimensional distribution profiles at organ and tissue scales (fluorescence diffuse optical tomography (fDOT), fluorescence-mediated molecular tomography (FMT)) 159, 160. Most importantly, the possibility for high spatial and temporal resolution imaging enables tissue and (sub-)cellular scale imaging in preclinical set-ups via intravital microscopy (IVM) (confocal laser scanning microscopy (CLSM) and two-photon / multiphoton microscopy (MPM)) 161, 162. Real-time *in vivo* optical imaging modalities are steadily substituting “old-school” *ex vivo* methodology - “dead mice tell too few tales” 163 - and, preclinically, they establish high-resolution alternatives to conventional clinical imaging modalities. In addition, supplementary *ex vivo* / *in vitro* optical imaging techniques can provide supportive structural and functional information (immunohistochemistry (IHC) on tissue slices, electron microscopy (EM), flow cytometry imaging). Optical imaging is widely used in the development and evaluation of nanotherapies: molecular imaging helps unravel nanoparticles' complex *in vivo* fate 164, 165, while development of nanoparticles with multimodal-imaging potential 166-170 and state-of-the-art fluorescence-labelling strategies 171, 172 increase the amount and quality of information.

### Whole body fluorescence imaging on organism and organ / tumour scale

**Figure 2:**
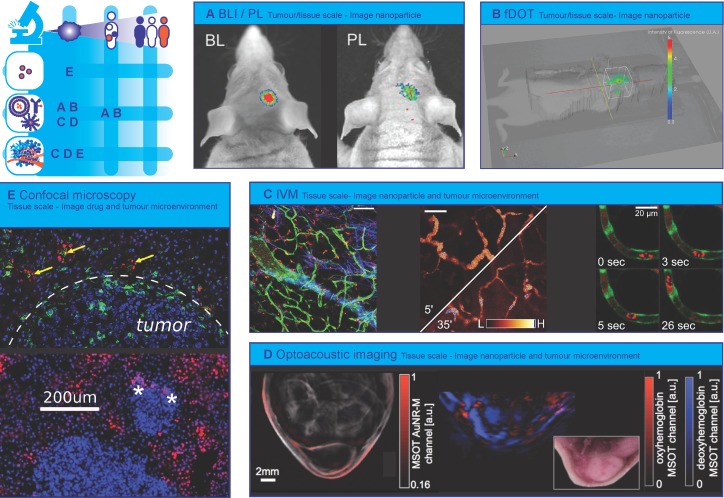
** Optical technologies to image spatial heterogeneity of nanomedicine. (A)** Combination of bioluminescence imaging (BLI) of luciferase expressing glioblastoma and photoluminescence (PL) imaging of theranostic photonic nanoparticles to verify nanoparticle tumour targeting efficacy. *(Adapted with permission from 181, copyright 2016 Wiley)*. **(B)** 3D fluorescence-enhanced diffuse optical tomography (fDOT) image after injection of NIR-decorated nanoparticles in tumour-bearing mouse. *(Adapted with permission from 173 copyright 2012 SPIE Digital Library)*. **(C)** Representative examples of real-time intravital microscopy (IVM) used to visualizing tumour microenvironment and track nanoparticles. The combination of bright-field illumination, non-linear optical imaging effects, endogenous fluorescence, and i.v. administration of fluorescent dyes contribute to a high quality tumour microenvironment characterization. **Left**: Green fluorescent protein (GFP) expressing endothelium (green) in a TIE2GFP mouse, 70 KDa TMR-dextran positive TAM (red), collagen (blue). **Middle**: Rhodamine-labelled nanoemulsions, passive diffusion on inflamed tissue over 30 min. **Right**: Atto633-labelled Doxil-like liposomes in circulation (red blur within vessel) and phagocytosed by a slow-moving circulating immune cell (red blob), GFP expressing endothelium (green) on TIE2GFP mouse (green). Scale bars 100 μm (right: 20 μm).* (A.M. Sofias and S. Hak, unpublished data)*. **(D)** Multispectral Optoacoustic Tomography (MSOT) images of nude mouse with A2780 tumour **Left**: gold nanorod accumulation (overlaid in red) 24 hours after injection **Right**: MSOT images of oxyhemoglobin (red) and deoxyhemoglobin (blue) distribution visualizes vasculature. *(Adapted with permission from 277, copyright 2012 Radiological Society of North America (United States)).* **(E)** Heterogeneity of transport and structural properties of 4T1 breast cancer metastases in mouse liver. Several magnified metastases with different sizes and the red fluorescence of extravasated doxorubicin delivered by PEGylated liposomal doxorubicin (PLD) and colocalizing (yellow arrows) with Kupffer cells (green) outside tumours; stars denote doxorubicin fluorescence in tumours, the white-dashed line indicates the tumour *boundary (Adapted with permission from 225, copyright 2018 Elsevier).*

Traditionally, macroscopic optical imaging modalities are used in preclinical small animal experimental procedures as alternatives to conventional non-terminal / non-invasive whole-body imaging modalities (PET/SPECT, MRI, CT). BLI, one of the most commonly used optical imaging techniques, allows real-time detection of protein-derived native light emission (Fig. 2A). Even though the required genetic engineering (transfection of cancer cell lines, transgenic animals) makes the technique inapplicable to wild type tumours 173, BLI remains a fast and user-friendly option to evaluate nanotherapy efficacy based on the endogenous luminescence of tumours 174-180 to verify nanoparticles' diagnostic or theranostic potential 168, 181, 182, and to combine with other imaging approaches 183. Another extensively used preclinical imaging technique is whole body fluorescence imaging (FLI), which requires the administration of fluorescent nanoparticles or molecules (Fig. 2A). Whole body FLI allows for a two dimensional organism and organ/tumour scale evaluation of nanotherapy spatial heterogeneity. Researchers use FLI to define nanoparticles' *in vivo* release profile 184, to monitor nanoparticle tumour accumulation 185-189, to determine how specific structural characteristics of nanoparticles alter their tumour accumulation 186, to examine nanoparticles theranostic potential 170, 190-192, to evaluate the performance of nanoparticles as potential single- 193-195 or multimodal 168, 170, 196, 197 imaging probes. Despite the fact that BLI and FLI can be used to delineate solid tumours and detect fluorescent nanoparticles respectively, they fail to provide three-dimensional and deep-tissue information. This disadvantage can be surpassed by integrating fluorescence-driven tomographic techniques 159. fDOT 173 and fluorescence molecular tomography hybridized with computed tomography (FMT-CT) 198-200 in combination with Near-infrared (NIR-) decorated nanoparticles provide additional three-dimensional spatial information (Fig. 2B).

### Intravital microscopy on tissue and (sub) cellular scale

Recent advancements in molecular imaging revealed the dark side of the field of nanomedicine. The scepticism about the EPR effect 39, the demonstration of low targeting efficiency towards tumour cells 201, and even, surprisingly, the incrimination of nanomaterials as metastasis mediators 202 denote that nanoparticle behaviour *in vivo* is highly complex. Therefore, a deeper understanding of *in vivo* behaviour, targeting mechanisms and nanoparticles' specific engagement with cell populations (tumour, stromal, endothelial, immune cells) is essential. Extensive use of real-time imaging techniques like IVM, could potentiate our efforts to characterize the tumour microenvironment on tissue and cellular scale (Fig. 2C) and design nanotherapies with predictable and desired physicochemical and immunobiological behaviour.

### Imaging the nanoparticle

Indeed, IVM can provide information regarding nanoparticles' extravasation, diffusion, and penetration into tumours (Fig. 2C). For such purpose, orange/red fluorescence-labelled nanoparticles are most commonly injected together with large molecular weight (e.g. 2 MDa) green fluorescence-labelled dextran to delineate vessels. This two-dye strategy was applied to confirm the silica nanoparticle-based delivery of small interfering ribonucleic acid (siRNA) cancer therapeutics to orthotopic MDA-MB-231 tumours 203. Similarly, extravasation of 100 nm long circulating liposomes into melanomas in presence / absence of Tumour Necrosis Factor (TNF) co-administration was evaluated. The IVM experiment revealed TNF-mediated vessel permeabilization that led to enhanced liposome extravasation. Unsurprisingly, the TNF-derived benefit was not observed for liposomes of larger size (400, 800 nm) 204, corroborating the realization that nanoparticles much larger than 100 nm cannot extravasate. Alongside qualitative visualization, IVM was utilized for semi-quantitative analysis comparing accumulation of nanoparticles in tumours versus healthy organs 205.

Interestingly, IVM has been used to identify differences in nanoparticle diffusion to tumour sites or nanoparticle tumour targeting, and to correlate them to different physicochemical properties, providing an excellent tool for head-to-head nanoparticle comparisons. The size-dependent diffusion of nanoparticles was studied after administration of a library of small fluorescent quantum dot nanoparticles with diameters of 12, 60 and 125 nm, revealing that 12 nm nanoparticles diffused twice as far in comparison to the largest size particles 206. Similarly, a size effect was found when attempting to target lymph node metastases with nanoparticles: of three nanoparticles with diameters of 30, 70, and 80 nm, only the smallest reached the metastasis 207. By comparing the studies that aimed to understand the importance of the nanoparticle size in *in vivo* behaviour, a clear pattern of deeper tissue penetration by smaller nanoparticles is revealed. In another study low (5 mol %) PEG surface density proved to contribute to a higher targeting specificity of arginylglycylaspartic acid (RGD) nanoparticles, than high (50 mol %) surface PEG density 208. A head-to-head comparison between the extravasation of a quantum dot and a nanotube sharing similar surface coating, surface area, and charge but different geometry (spherical vs cylindrical respectively) revealed shape-dependent and tumour-dependent extravasation patterns. Of three investigated tumour models, cylindrical single-walled carbon nanotubes were found to extravasate only markedly in a human glioblastoma tumour model, while spherical quantum dots extravasated only in a colon adenocarcinoma tumour model. Surprisingly, no extravasation of either nanoparticle was observed in an ovarian adenocarcinoma tumour model 209. Comparably, heterogeneity in extravasation patterns between these tumour models was found for RGD-decorated and control quantum dots 210. The importance of morphology was emphasized when plateloid-shaped microparticles were found to adhere more efficiently to tumour vasculature and exhibit a higher tumour to liver accumulation ratio than cylindroid microparticles 211.

### Imaging the tumour environment

In addition to addressing nanoparticle tumour targeting and accumulation profiles, IVM has been used to unravel more intricate interactions between nanoparticles and immune cells; an interesting option given the increasing appeal of cancer immunotherapy. One of the first studies providing real-time insight in the behaviour of TAM, developed magneto-fluorescent nanoparticles enabling the visualization of nanoparticle-labelled TAM on tumour/organ scale (MRI, FMT) and on tissue and cellular scale (IVM). Among other findings, TAM phagocytosed more nanoparticles than other myeloid cells, they were situated in close proximity to tumour cells and displayed low motility 212. Since nanoparticles tend to accumulate in TAMs, the hypothesis that an increase in TAM population within the tumour microenvironment would also increase nanoparticle accumulation was tested. Application of radiation proved to increase TAM / tumour cell ratio and confirmed this hypothesis: radiation enhanced the accumulation of liposomal doxorubicin in the tumour. IVM experiments showed that the enhanced accumulation was mediated by an increase in 'vascular bursts' (bursts of extravasation of nanoparticles into the tumour tissue) for which the presence of TAM and perivascular phagocytes was required 213. Of note, the verification that vascular bursts are a driving mechanism for enhanced accumulation of nanoparticles within a tumour, challenges the conventional EPR-effect theory about roughly homogeneously increased leakiness of tumour vasculature 214. Another immune cell-related variable that has been tested via IVM is the nanoparticle clearance from circulation. Myeloid immune cells proved to be a significant mediator of nanoparticle clearance, as nanoparticles injected in mice pre-treated with clodronate (which causes depletion of phagocytotic cells), circulated in a significantly higher amount in the blood 215.

Given that angiogenesis is a hallmark of cancer, the attention of intravital microscopy users has been directed particularly to nanoparticle-mediated vessel wall visualization and targeting. Already in the mid '00s, a successful attempt using vascular cell adhesion molecule 1 (VCAM-1) decorated nanoparticles paved the way for future success 216. Subsequently, the development of RGD-decorated multimodal (fluorescence and paramagnetic) quantum dots 217 or nanoemulsions 208 aimed to actively target the αvβ3 integrin receptor overexpressed by angiogenic endothelium. By comparing the above studies, we see that attaching a certain targeting peptide to a nanoparticle alters the nanoparticle's *in vivo* behaviour in a similar manner regardless of the selected nanomaterial, i.e., nanoemulsions versus quantum dots. The multimodal nature of nanoparticles allows for visualization of angiogenic endothelium and neovasculature on tumour/organ scale (MRI, FLI, BLI), tissue scale (IHC and IVM), and cellular scale (IVM) 208, 217. Of note, IVM strategies of visualizing nanoparticles and immune cells could be expanded outside the field of nanomedicine, for cancer cell imaging 218, which could be used as complementary technique in the analysis of liquid biopsies 219 and in the assessment of tumour heterogeneity 220.

The new mechanistic and molecular insights that were obtained through IVM procedures inspired researchers to develop IVM-specialized imaging agents. Biocompatible organic dots for MPM 221, magneto-fluorescent nanoparticles 222 and fluorescent nanoprobes that detect vascular permeability 223 are among representative examples of nanoparticles aiming to increase the information obtained by imaging.

### Optical imaging of drugs

Visualization of fluorescent-labelled nanoparticles and related aspects of the tumour microenvironment provide valuable information on therapy heterogeneity in nanomedicine. Imaging the administered drug itself would complete the picture. However, direct imaging of drugs remains elusive. In this respect, inherently fluorescent drugs are convenient 224, 225, and some chemotherapeutic drugs relevant to cancer research possess fluorescent properties (i.e. doxorubicin, mitoxantrone, irinotecan) 226. The application fluorescent drugs is nicely illustrated by a study that showed colocalization of fluorescent doxorubicin with Kupffer cells outside of tumours in a liver metastasis mouse model after treatment with PEGylated liposomal doxorubicin (Fig. 2E) 225. Another interesting study from our institution used ex-vivo fluorescence microscopy to quantify tumour tissue doxorubicin concentration and heterogeneity of doxorubicin distribution after treatment of mice with doxorubicin, PEGylated liposomal doxorubicin (Doxil) and temperature-sensitive doxorubicin liposomes (ThermoDox) at three different dosages. Heterogeneity in doxorubicin distribution was visualized on tissue scale and could be compared spatially to heterogeneous vessel perfusion, hypoxia and dividing cell fraction in the tumour microenvironment 227. However, *in vivo* imaging of inherently fluorescent drugs is hampered by their relatively low fluorescence quantum yield, which limits their detectability at therapeutic concentrations. Another approach could be the conjugation of fluorescent molecules to the drugs that are carried by nanoparticles, despite the fact that this could result into alternation of their properties. The conjugation of fluorescent dyes to macromolecular drugs has been successfully applied before 87, 96, 228. In these studies the drugs had a significantly higher molecular weight than the conjugated fluorescent dye, which made their biodistribution properties, targeting specificity and efficacy less likely to be compromised by the dye. Fluorescently labelled therapeutic antibodies have already been administered to patients in early clinical studies 229-231.

### Tissue optical clearing

Utilization of fluorescent molecules is often restricted by factors such limited imaging depth. Therefore, more sophisticated *ex vivo* techniques like the tissue optical clearing strategies 232 have been developed to surpass these limitations 233 by reversing the tissue opacity 234. The application of such a methodology has been successfully applied to 3D cell spheres 235, tissue samples 236, 237, intact organs 238, 239 and even entire organisms 240. Even though most of the conducted research is performed in soft tissues and organs, e.g., the brain, the tissue optical clearing strategies appear an appealing methodology for visualizing tissues in which fluorescence signal is heavily scattered, such as the dense connective tissue 241. Tissue clearing applications provide high quality 3D information and improved mapping of the tissue environment which is useful to investigate the nanoparticle 242-244 and drug 228 distribution at tissue, organ, and organism scale. Additionally, tissue clearing has been used as a tool to study the heterogeneity of immune cell infiltration and therapeutic response in tumour models 245. Besides its pre-clinical use, tissue clearing methodology has been applied for microscopic assessment of clinical specimens 246.

### Clinical translation of optical imaging

Due to the limitations in tissue penetration and size of the imageable subject, *in vivo* optical imaging is mainly constricted to preclinical applications. Clinically, optical imaging is of course widely used on *ex vivo* biopsy or surgical samples. Although this consists mostly of immune histochemistry, some work has been done to complement this with fluorescent imaging 247, 248. To our knowledge, fluorescent imaging has not yet been applied in clinical trials using nanomedicine. However, progress has been made towards translation of the use of silica nanoparticles for intra-operative sentinel node and tumour detection 249, 250. In the future ex-vivo analysis of patient biopsies or surgical samples during clinical nanomedicine trials could provide detailed information on therapy heterogeneity on the tissue scale by visualizing nanoparticles, tumour microenvironment and perhaps fluorescent drugs. However, non-invasive in-vivo techniques will probably remain more appealing. Apart from nanomedicine, the clinical use of optical imaging is mostly complementary, with primary focus on intraoperative imaging 248, 251-253 and fluorescence-guided diagnosis 248, 254.

### Optoacoustic imaging

Optoacoustic (photoacoustic) imaging is an emerging hybrid technique that combines the benefits of US and optical techniques, i.e. deep imaging depth, high spatial resolution and high contrast 255. In optoacoustic imaging energy emitted by a pulsed laser source is absorbed by tissue causing its thermoelastic expansion, which generates ultrasound waves that can be detected with conventional ultrasound transducers 256. The spatial resolution and imaging depth can be adapted to the scale of the preferred application domain, ranging from cellular substructures to organs with the same type of contrast 257. Optoacoustic signal is mainly provided by endogenous molecules, such as haemoglobin (Hb), melanin, lipids, and collagen, or exogenous contrast agents such as small-molecule dyes, gold nanoparticles and liposomes 258, 259.

### Imaging the tumour environment

In the oncology domain the endogenous contrast is typically used to study tumour vasculature 260, 261 and oxygenation status (Hb) 262, 263 at cellular scale 264 as well as at tissue scale 265. Whereas targeted exogenous contras agents enable the readout of a specific biological entity or process such as Epidermal Growth Factor Receptor (EGFR) expression 266 or matrix metalloproteinase activity 267. By using multiple wavelength illumination (i.e. Multispectral Optoacoustic Tomography (MSOT)) it is possible to differentiate the contribution of different contrast agents and analyse their concentration and distribution simultaneously. In the work of Tomaszewski et al. MSOT imaging of endogenous contrast (i.e. signals from oxy- and deoxyhaemoglobin) and exogenous contrast (signals from the FDA approved organic dye indocyanine green (ICG)) allowed for the non-invasive assessment of tumour vascular function, hypoxia, and necrosis revealing a complex, yet consistent network of relationships in the tumour vascular microenvironment 262. Okumura et al., in turn, showed the potential of photoacoustic imaging coupled with ICG for evaluating changes in tumour vascular permeability associated with antiangiogenic therapy 268. ICG rapidly binds to albumin in plasma, becoming a macromolecule that is not able to extravasate from vessels with intact endothelium. Reduced vessel permeability after anti-VEGF therapy, perceived as photoacoustic signal decrease in the tumour, was detected before inhibition of tumour growth indicating the potential of optoacoustic imaging as early marker of therapy response. Reporter gene products such as β-galactosidase 269, tyrosinase 270, 271 and fluorescent proteins 272 have also been used to produce contrast for optoacoustic imaging. Recently, Peters et al. introduced a new approach for creating optoacoustic imaging contrast by injecting phototrophic purple bacteria into tumours, which allowed them to monitor *in vivo* spatiotemporal changes of macrophage activity 273. The spatiotemporal distribution and activity of macrophages are very relevant for nanomedicine since macrophages are increasingly being used for targeting nanoparticles towards tumour cells 274.

### Imaging the nanoparticle

Nanocarriers not only serve as optoacoustic contrast agent, but can also act as vehicles for drugs. Several nanoparticles have been loaded with drugs and combined with optoacoustic imaging for non-invasive and real-time monitoring of biodistribution and pharmacokinetics 275, 276. Herzog et al. used the MSOT approach to investigate the accumulation over time of long-circulating gold nanorods as well as intratumoural patterns of hemoglobin oxygenation to demonstrate imaging of the EPR effect (Fig. 2D). Higher nanorod accumulation was seen in the tumour model with a higher fraction of deoxygenated haemoglobin, although the underlying mechanism is still unclear 277. The work by Song et al. illustrates nicely how MSOT is applied for whole-body visualization of the nanocarrier-based drugs distribution as well as the blood vessels in mice. They demonstrated that the distribution of platinum containing nanoparticles in tumours is highly vascularity-dependent, and could only access the peripheral region of the tumours 278. Similarly, Kim et al. used bioconjugated gold nanocages as a contrast agent for quantitative molecular optoacoustic tomography of melanomas and surrounding blood vessels at microscopic scale *in vivo* 279. These gold nanocages have already been used for triggered drug delivery 280. Another interesting approach is to use pulsed laser irradiation, intrinsically part of optoacoustic instrumentation, as a stimulus for triggered drug release 281. Here, low-intensity laser irradiation was used for photoacoustic imaging, while high-intensity laser irradiation induced the vaporization of perfluorohexane loaded in the nanoparticle and triggering the fast release of the co-loaded drug paclitaxel.

### Imaging the drug

In order to monitor the drugs themselves using optoacoustic imaging they should exhibit NIR-absorbing properties. Unfortunately, few if any clinically prescribed drugs have strong intrinsic absorption in the NIR. As an alternative, small molecule NIR dyes are co-loaded with drugs of interest to monitor drug release and distribution 282. An alternative approach for monitoring drug release was recently proposed by Yang et al. 283. They developed a multifunctional nanotheranostic platform consisting of two optoacoustic imaging probes that allowed for concurrent non-invasive real-time ratiometric optoacoustic imaging of acidic tumour pH and monitoring of pH-induced drug release in living mice.^284^

### Photothermal and photodynamic therapy

Optical and optoacoustic imaging have frequently been combined with photothermal therapy (PTT 170, 187, 190, 285) and photodynamic therapy (PDT 168, 188, 189) so that the NIR excitation can be used for both imaging and therapy. The recent progress in this field was excellently reviewed by Zhu et al. 286. In PDT a photosensitizer is administered, which is subsequently activated by external light. In PTT nanoparticles generate heat upon laser light excitation. Nanoparticles (e.g. gold nanoparticles) can act as photothermal agents while simultaneously delivering photosensitizing agents 287. Clinical trials using these therapies have already been performed 288 and imaging the distribution of photodynamic and photothermal agents could help towards further clinical translation.

### Mass Spectrometry Imaging

Mass Spectrometry Imaging (MSI) is a label free, multiplex technique that is used to visualize the molecular distribution of endogenous compounds such as metabolites^289^, lipids 290, 291, proteins and peptides 292-294, as well as drugs 295, 296 and drug delivery systems 297 in biological tissues. MSI therefore has the ability to collect not only drug distribution data but also endogenous compound information related to drug-induced efficacy and toxicity on tissue and cellular scale. This technique is increasingly being used in the pharmaceutical research and development pipeline and has demonstrated its utility from early stage drug discovery to preclinical development and clinical evaluation of tumour response to treatment. MSI is used for i) localizing and quantifying drug and metabolite levels (pharmacokinetics) to study efficacy 295, ii) assessing off target drug accumulation to study toxicity 298, 299, and iii) detecting endogenous biomarkers (pharmacodynamics) for predicting and evaluating treatment response 300.

In MSI spatially defined desorption/ionization methods are used to collect sequentially mass spectra from a small region (pixel) of a tissue sample. Among the multitude of surface sampling techniques, matrix-assisted laser desorption/ionisation (MALDI) uses a laser beam for desorption/ionization of tissue-representative molecules co-crystalized in a solidified matrix; while desorption electrospray ionization (DESI) makes use of an electrically charged solvent spray and in secondary ion mass spectrometry (SIMS), a beam of high energy primary ions (e.g. Ar+, Ga+, In+) is used to release secondary ions from the sample surface. In contrast to MALDI and SIMS where the sample is analysed under vacuum, DESI is non-destructive and performed at atmospheric pressure, which renders the technique more user friendly and through appropriate solvent selection, more tuneable to increase selectivity and/or sensitivity. Depending on the ionization method used spatial resolution, sensitivity, and the molecules that can be analysed change. Mostly, MALDI is used at 10 to 20 µm spatial resolution, whilst DESI resolution spans 50 µm till 200 µm and SIMS allows for sub-µm resolution. Sensitivity wise DESI outperforms MALDI and SIMS, partly due to increased pixel size (see spatial resolution) and partly due to improved ionization efficiency. Although sensitivity is determined by e.g. physiochemical properties of the compound, typically one requires low µg/g concentrations in the case of DESI and approx. 20 µg/g tissue for detection by MALDI and SIMS.

### Imaging the drug

**Figure 3:**
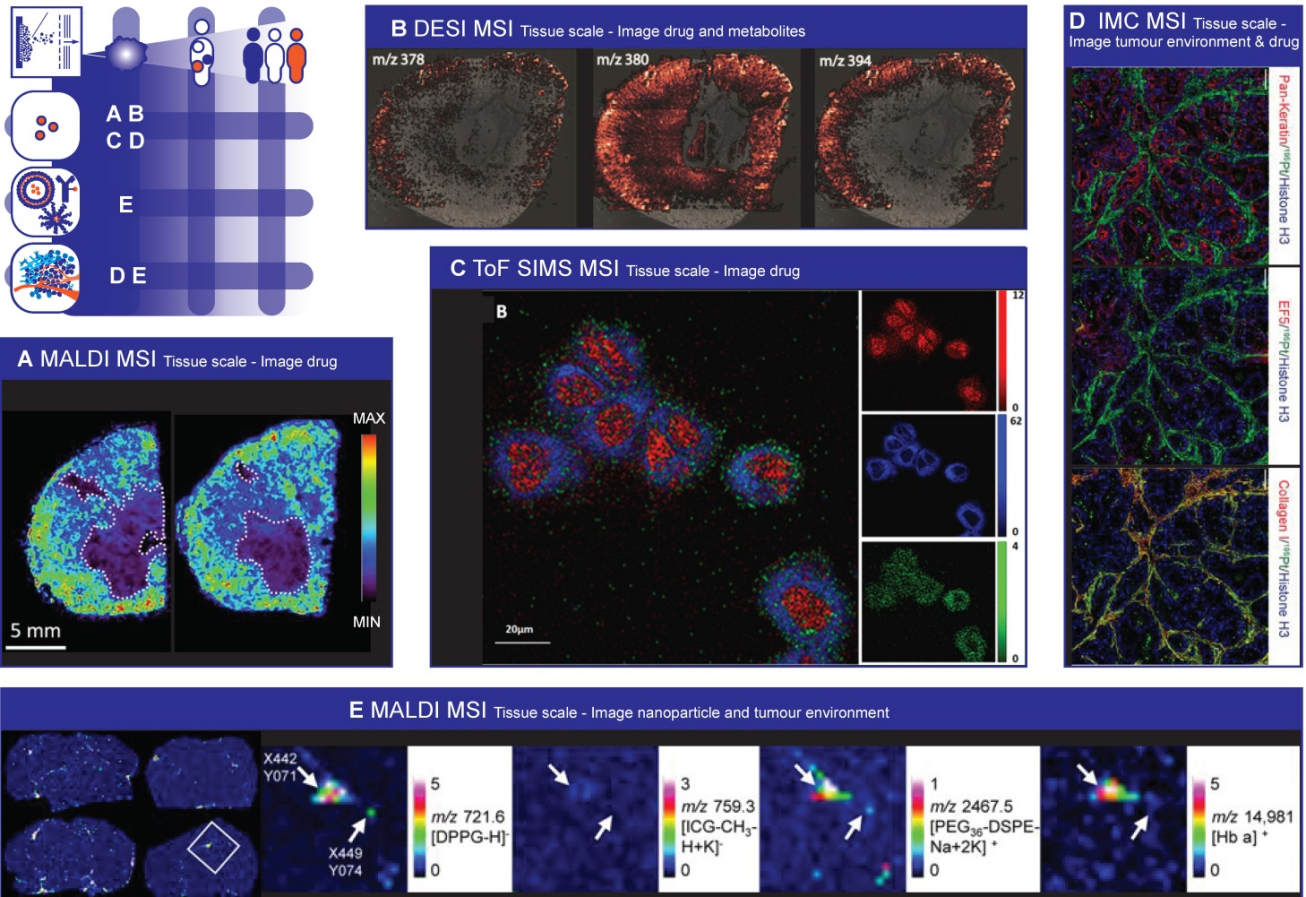
** Mass Spectrometry Imaging to image spatial heterogeneity of nanomedicine. (A)** Paclitaxel distribution by MALDI MSI. Necrotic areas, highlighted with dashed lines, are those were there is the lower drug signal. (*Adapted with permission from 307, copyright 2016 Nature Research).* **(B)** DESI image overlay representing the spatial distribution of the drug compound (*m/z* 378) and its most abundant metabolites (*m/z* 380 and 394) in a tissue section of a formalin fixed frozen rabbit kidney. (*Adapted with permission from 313, copyright 2016 Springer US).* **(C)** ToF SIMS 2D images of 3D data acquired in higher spatial resolution mode from HeLa cells completely consumed by the argon cluster source during analysis. The cells were incubated for 2 h with 9.7 nmol/mL amiodarone hydrochloride. Composite image where red represents ribose *m/z* 81, blue shows the signal from the phosphatidylcholine lipid fragment (m/z 184), and green shows the amiodarone signal, [M + H]+ (*m/z* 646).* (Adapted with permission from 317, copyright 2017 American Chemical Society).* **(D)** Cisplatin effects on tumour proliferation, DNA damage and cisplatin distribution in the tumour. Representative Pan-Keratin, EF5, Collagen I, 195Pt, and Histone H3 images of cisplatin-treated (40 mg/kg for 24 h) mice with OCIP28 patient derived xenografts. Scale bar = 100 μm.* (Adapted with permission from 321, copyright 2016 Nature Research).* **(E)** MALDI MSI images performed on brain slices of mice that were dosed with liposomes. **Four images on the left**: Half of the mice were perfused before being sacrificed (right panels) to reduce the remaining blood in the tissue. MALDI images of liposomal marker 1,2-dipalmitoyl-sn-glycero-3-phosphoglycerol (DPPG) and indocyanine green (ICG) were acquired in reflector negative ion mode, of PEG36-DSPE in reflector positive mode and of Hb α chain in linear positive mode. DPPG, ICG and 1,2-distearoyl-sn-glycero-3-phosphoethanolamine conjugated with monodisperse polyethylene glycol (PEG36-DSPE) were measured with 4-Phenyl-α-cyanocinnamic acid amide (PhCCAA) MALDI matrix. Hb was detected after delipidation and 2,5-dihydroxybenzoic acid (sDHB) deposition on the same tissue region. **Magnifications**: MALDI-MS images of the boxed parts marked in perfused brain in pixels indicated by an arrow shows the co-localisation of the liposomal components and hemoglobin at pixel X442 Y071 and the absence of HB at pixel X449 Y074. *(Adapted with permission from 324, copyright 2016 Nature Research)*

The number of drugs that have been detected using MSI is extensive, ranging from anti-cancer drugs (e.g. paclitaxel 301, sunitinib 302, doxorubicin 303), antibiotics (moxifloxacin 304, polymyxin 298), beta blocker propranolol 305 and antipsychotic drug olanzapine 306. MALDI and DESI are mainly used to study the drug distribution at tissue scale. MALDI MSI images for example showed clearly that the distribution of paclitaxel distribution is very heterogeneous and depends on the histopathological characteristics of the different tumour models investigated (figure 3A) 307. High performance liquid chromatography (HPLC) analysis of tumour homogenates was not able to detect the heterogeneous drug distribution in tumour sections. The same group also showed that the anti-angiogenic agent bevacizumab induced changes in the tumour microenvironment (i.e. more uniform distribution of vessels and less necrosis). Bevacizumab led to a more homogeneous distribution of paclitaxel and even though the total tumour paclitaxel concentration was lower, anti-tumour activity was greater 308. In a comparable study, Torok et al. explored the effect of the intratumoural concentration and distribution of five receptor tyrosine kinase inhibitors on their anti-vascular and anti-tumour activities 309. They demonstrated that limited tumour tissue drug penetration was the primary source of resistance to angiogenesis inhibitors. Both studies clearly show the impact of drug distribution on pharmacological responses and demonstrate the potential of MALDI-MSI to predict the efficacy of unlabelled small molecule drugs in malignant tissue.

Unlike whole-body autoradiography, which is the standard for quantitative assessment of drug distribution, MSI can detect the parent drug and metabolites simultaneously in a single experiment, without having to label the drug 310, 311. For example Liu et al. imaged the time-dependent and concentration-dependent permeability and metabolism of irinotecan in tumour organoids. They discovered that the active metabolite SN-38 did not co-localize well with the parent drug irinotecan and the inactive metabolite SN-38G, which may lead to therapy heterogeneity 312. Bruinen et al. were able to find out using MALDI and DESI that precipitation of crystal-like structures in the cortex of rabbit kidney, which were assumed to cause the renal toxicity, were mainly composed of metabolites and relatively little parent drug (figure 3B) 313. In another example, Groseclose et al. 314 reported on the nephrotoxicity of dabrafenib, an approved drug for treatment of specific tumours in adults. Pre-clinical studies showed renal pathogenesis due to obstructive nephropathy in juvenile rats. MSI allowed for spatial analysis of DAB and its metabolites and determination of the chemical composition of the renal deposits. It showed that the deposits were dabrafenib- and dabrafenib metabolite-free and they were merely composed of calcium phosphate. Hence a better risk assessment for pediatric treatment with dabrafenib was performed.

So far, MALDI-MSI cannot yet match the spatial resolution of established methods for intracellular imaging such electron microscopy. However, using SIMS it is possible to map the distribution of drugs within individual cells 315. For example, SIMS was used to localize the drug amiodarone at therapeutic dosing concentrations in four different cell types (figure 3C) 316, 317. SIMS was also employed to study the intracellular accumulation of two drugs (p-boronophenylalanine (BPA) and sodium borocaptate (BSH)) used for boron neutron capture therapy 318. By labelling each drug with a different boron isotope (i.e. 10BPA and 11BSH), they were able to image the subcellular distribution of both drugs independently in the same cell. In a recent paper by Vanbellingen et al. the distribution of the B-cell lymphoma 2 (Bcl-2) inhibitor ABT-737 was studied in a treated A-172 human glioblastoma cell line 319. They were able to visualize the drug and some endogenous markers on the (sub-)surface of the cells with high spatial (~250 nm) and high mass resolution (m/Δm ~10,000), and absence in the nucleus, confirming site of action.

An alternative and novel MSI technique for imaging drug distribution at subcellular resolution, the so-called imaging mass cytometry (IMC), was introduced in the life sciences by Giessen et al. in 2014 320. IMC is based on laser ablation inductively coupled plasma mass spectrometry (LA-ICP-MS), and provides capability to either analyse drugs containing metal ions, like e.g. cisplatin, or use antibodies labelled with a polymer containing (rare-earth) metals (e.g. Europium, Gadolinium, Gold, Platinum). Because these metals all have distinct isotopic patterns and are absent in biological specimens, they can be quantified with high precision. Next the use of laser ablation offers the possibility to excise tissue sample of (sub-)micron size, providing an order improved spatial resolution. Using this technique Chang et al. imaged the platinum distribution at subcellular resolution (1 µm) in patient-derived pancreatic cancer xenograft-bearing mice treated with cisplatin, revealing extensive binding of platinum to collagen fibres in both tumour and normal mouse tissues (Figure 3D) 321. Theiner and coworkers also employed LA-ICP-MS to localize platinum in the kidney in mice treated with three different Pt-containing drugs. The imaging data revealed that the drugs were mostly located outside of the malignant parts of the samples. This clearly demonstrates that determining average Pt concentrations might overestimate drug uptake and cause misleading conclusions on therapy efficacy 322.

### Imaging the nanoparticle

MSI also provides the opportunity to image nanocarriers, such as lipid- and metal-based nanoparticles. Typically nanoparticles are labelled or loaded with a radioactive or fluorescent probe in order to follow the *in vivo* fate after administration. However, this requires additional chemical development and the introduction of the probe may influence the biodistribution of the nanoparticle. Recently, Zandanel et al. showed that MALDI-MSI allows for the simultaneous visualization of the polymeric nanoparticle, the encapsulated drug (doxorubicin) and its metabolite (doxorubicinol) in treated mouse liver 323. Unfortunately, they didn't show the co-localization of the nanoparticle and the drug in the same tissue section. Fülöp et al. exploited the multiplex nature of MSI even further by determining the spatial distribution and integrity of drug-loaded liposomes in tissue with a single label-free measurement 324. By imaging two lipids (DPPG and PEG36-DSPE) incorporated in the liposomal bi-layer they were able to visualize the liposome distribution, and in addition they could interrogate the integrity of the liposomes by looking at the co-localization of the two lipid markers (figure 3E) 324. Furthermore, they examined the presence of remaining blood in the same tissue slice by MALDI imaging of hemoglobin, which allowed determining the localization of the liposomes with respect to the blood vessels.

Xue et al. developed an MSI method that enabled not only the visualization, but also the quantification of the *in situ* drug (doxorubicin) release from molybdenum disulphide (MoS2) nanosheets 325. The quantification of the drug release was done calculating the intensity ratios for doxorubicin and MoS2 signals. In two mouse tumour models (H22 and 4T1) they observed that the accumulation of drug-loaded MoS2 nanosheets was high in the spleen and liver, but the tumour tissue accumulation was much lower. However, the highest drug release from carriers was observed in tumour tissue, which was ascribed to higher drug dissociation extent due to the acidic tumour microenvironment.

### Imaging the tumour environment

As indicated earlier, mass spectrometry offers unique capabilities for untargeted exploration of biological samples and provides simultaneous information of the distribution of the drug, the nanoparticle and endogenous compounds such as metabolites, lipids and proteins. Therefore, MSI can be used to detect biomarkers associated with disease, molecular changes due to drug treatment and tumour components limiting nanomedicine distribution and effect or augmenting off-target effects.

Several studies report that the heterogeneous distribution of lipids and proteins could reflect the effect of therapy and/or could be used as prognostic/predictive marker for outcome. A very nice example of identifying and using endogenous proteomic profiles for distinguishing between responders and non-responders to chemotherapy for oesophageal adenocarcinoma is given by Aichler et al. 326. Here they selected a series of proteins in pre-therapeutic biopsies, which were identified through liquid chromatography mass spectrometry (LC-MS) analysis and investigated for functional relevance in-vitro. They identified a proteomic signature that was correlated with pre-existing defects in the mitochondrial respiratory chain complexes of cancer cells and was predictive for response to neoadjuvant cisplatin chemotherapy. Yanagisawa et al. reported for the first time in 2003 the ability of MALDI-MSI to generate proteomics patterns of tumour subsets in non-small-cell lung cancer 327. They showed that protein profiles obtained from tumour tissue samples obtained during surgery could be used to accurately classify tumours and stratify patients into groups associated to poor or good prognosis. Bauer et al. employed MALDI-MSI to identify protein markers differentially expressed in tumour biopsies from patients displaying complete pathological response (pCR) and non-complete pathological response after neoadjuvant paclitaxel/radiation treatment for breast cancer 292. Proteomic profiling of liver tissue using MALDI-MSI was also used to compare toxicity of hollow CuS nanoparticles and hollow gold nanospheres after intravenous administration in mice 328.

Also tumour hypoxia is investigated by many groups since it is associated with tumour aggressiveness and resistance to cancer treatment. Manscini et al. used MALDI-MSI to simultaneously detect pimonidazole, a clinically used hypoxia marker, its metabolites and associated biomolecules in a single experiment 329. They detected several endogenous species that co-localized with the hypoxic regions. Interestingly, these identified species are known to be involved in hypoxia or metabolic reprogramming in cancer, although their specific roles remain to be elucidated. Masaki et al. studied the distribution of ^18^F-fluoromisonidazole (FMISO), a widely used PET hypoxia imaging probe 330. The mass spectrometry images showed that FMISO and its metabolites were nearly homogenously distributed in the tumour and did not correlate with the radioactivity distribution. However, they identified a glutathione conjugate of amino-FMISO which did co-localise with the radioactive signals and was involved in FMISO accumulation in hypoxic tumour tissues.

Interestingly, most MSI studies that investigate drug distributions do not yet exploit the multiplex capabilities of MSI. Instead of using the wealth of information on endogenous molecule distributions (lipids, proteins, hemoglobin) already available in the acquired MSI image they superimpose the drug MSI images with standard H&E stained tissue images and/or various immunohistochemistry (IHC) images. As most of these IHC images are specific to a certain protein, this can be a laborious and time consuming effort. Moreover, correlating drug distribution with the distribution of these endogenous molecules would allow for non-supervised investigations to discover new factors that impair drug transport in tumour tissue and could be used as biomarker for prognosis and therapy response prediction.

## Discussion/ Conclusion

Spatial heterogeneity in nanoparticle distribution occurs at all scales and can reduce nanotherapy efficacy. A wide range of imaging modalities help visualize nanoparticle distribution or factors contributing to heterogeneous distribution, by imaging the drug, the nanoparticle and the tumour environment.

### Imaging scale

When selecting imaging modalities, researchers need to take into account the desired imaging scale. Non-invasive clinical imaging methods provide three-dimensional information of the intact body on patient, organ and tumour scale, and recent developments, such as super-resolution ultrasound, have broadened their application to provide even tissue scale information. Optical imaging modalities have a superior spatial resolution, procuring images on the tissue and cellular scale, and in the preclinical setting they can provide (sometimes non-invasive) organism and organ scale information as well. Besides that, *in vivo* applications and intravital techniques offer the possibility to visualize dynamic processes. Clinical use of optical imaging is still hampered by the limited penetration depth and therefore requires tissue sampling or intraoperative use. MSI provides information on tissue and even cellular scale. As it requires a tissue specimen it is inherently an invasive method, regardless of preclinical or clinical use. Due to this limitation, the technique is most suitable and currently most used in the preclinical setting, where an entire tumour or even the whole animal can be analysed at once. In contrast, in the clinic MSI approaches will most likely follow a workflow similar to standard histopathology. Emerging technologies allow for more accurate tissue sampling using intra-procedural multimodality imaging during biopsies 331, 332. Whenever biopsies are used sampling error is a disadvantage (just as it is for histopathology evaluations) and the technique is therefore less suitable for imaging on organ or tumour scale. Nonetheless, implementation of MSI in clinical research (especially when tissue samples are collected anyway) is feasible and adds a wealth of molecule distribution data on tissue scale. As such, MSI is increasingly becoming an established tool in clinical and pharmaceutical studies.

### Intrinsic versus extrinsic contrast

Nuclear imaging methods and most optical imaging methods require labelling of nanoparticles, drugs or aspects of the tumour environment, which has some disadvantages. First, the stability of the link between a label and a nanoparticle determines its usefulness in tracking the nanoparticle, for one could be imaging the label on itself after disconnection from the nanoparticle. Second, attaching a label could change the pharmacokinetic and drug release properties of the nanoparticle leading to erroneous prediction of the distribution of an unlabelled equivalent. Third, administering a labelled version of a previously approved therapeutic nanoparticle or drug could cause additional toxicity and obtaining clinical approval is cumbersome and costly. Fourth, administration of radiolabelled theranostic nanoparticles may decrease the effect of subsequent therapeutic administration through the ABC phenomenon 333. MSI provides a label-free alternative to obtain detailed tissue and cellular scale information on drugs, nanoparticles and the tumour microenvironment simultaneously.

### Multimodal imaging

The integration of imaging data collected from multimodality techniques offers a unique opportunity to combine information related to drug and nanoparticle distribution and tumour environment on multiple scales and provide synergistic advantages over the use of a single modality.

Clinical imaging modalities are generally combined to merge functional (SPECT, PET) with anatomical information (CT, MR) collected on the same scale. A range of nanoparticles for multimodality imaging have been developed 169, 196, 197, 334-336. On tissue and cellular scale, successful examples have shown complementarity between optical imaging and MSI techniques 337. Fluorescence and MSI have been combined to characterize local drug release and map unlabelled therapeutic drug distribution 311. MSI enabled monitoring of the drug and related metabolites, which were impossible to differentiate with solely fluorescence. The combination of MSI and IMC with IHC and fluorescence *in situ* hybridization (FISH), creates a new dimension to molecular pathology. Now, drug levels within tumour regions can be correlated to different degrees of vascularization of the tumour - highlighted with specific vasculature staining 338. One of the reported limitations is the impossibility to perform all analyses on the same tissue section. As a consequence and because a tumour is such a heterogeneous system, it is highly probable that two consecutive sections have different morphology / molecular content, which is then difficult to correlate accurately.

To achieve multiscale information, optical imaging and MSI have been paired with clinical imaging 59, 169, 183, 196, 197, 339. Using dual fluorescent and MRI probes, the high sensitivity of fluorescent imaging is complemented with MRI's ability for deep-tissue penetration and high spatial and temporal resolution 340, 341.

A challenge remains the integration of molecular information provided by 2D / *ex vivo* MSI or optical imaging with 3D / *in vivo* images, such as those generated with SPECT, PET, CT or MRI. This challenge is inherent to the fact that the images are acquired at different scales, can be subjected to different deformations, and lack common fiducial markers. A first step towards combining two-dimensional MSI with three-dimensional MRI is the ability to monitor MRI contrast agents with MSI, which was demonstrated by Tata et al. 342. They used DESI-MSI to monitor Gadoteridol, to characterize intratumoural heterogeneity and further guide delineation of tumour margins. Coregistration of MSI with other modalities is promising to bridge the gap between the different scales 343-347. It is worth mentioning that the route towards user-friendly automated methods that are needed to integrate these methods in a routine (clinical) workflow is still long. Nonetheless, multiscale and multi-aspect (i.e. nanoparticle, drug and environment) data is believed to open new doors to improve the characterization of spatially heterogeneous distribution and heterogeneous effect and could greatly contribute in the development of new and more effective nanotherapies.

### Role of imaging for clinical translation

Integration of imaging techniques in preclinical lab practice has contributed to an improved understanding of the *in vivo* behaviour of nanoparticles. Additionally, it has led to valuable information on how specific characteristics within the tumour environment can affect nanotherapy outcomes. Continued application of these imaging approaches, and especially the combination of different techniques, will further strengthen our understanding of therapy heterogeneity on organism, tissue, cellular and even subcellular scale. Meanwhile the role of imaging for monitoring PK and biodistribution is well established in research and development stage; however in the clinical setting it is not yet adopted. Clinically, spatial heterogeneity distribution of therapeutic nanoparticles, poses the threats of under- and overtreatment. Despite the fact that many studies have shown that drug concentration does not correspond with tissue drug levels, phase I and II clinical trials mainly rely on blood samples and spatially sparse biopsies to measure PK on patient scale and biodistribution on organ scale. Imaging drugs or nanoparticles in clinical studies is still rare and often no information is acquired about spatial heterogeneity of nanomedicine on organ, tumour or tissue scale. However imaging drug/nanoparticle distribution can help predict treatment effect and therefore select which patients will benefit most and in which patients a therapy adjustment or a combination therapy that reduces heterogeneity are warranted. Combining imaging of nanoparticle and drug distribution with imaging of tumour environment characteristics or early response indicators promises to help personalize treatment further. Currently, only optical imaging is commonly used to investigate, in the preclinical setting, the interplay between environment, nanoparticle and drug. It is our hope and recommendation that non-invasive clinical imaging and MSI will play a central role in future preclinical and clinical research on the interaction of drug, nanoparticle and environment. Choosing and combining imaging modalities wisely will lay the foundation for successful future nanotherapies.

## Figures and Tables

**Table 1:** Comparison of modalities to image spatial heterogeneity of nanomedicine.

Modality		Drug	Nanoparticle	Environment	Spatial resolution	Tempor. res.	Imaging depth	Strengths	Limitations
Non-invasive clinical imaging methods	PET / SPECT	Drug labelled with radioactive tracer (e.g. ^11^C, ^18^F and ^123^I).	NP labelled with radioactive tracer (e.g. ^64^Cu and ^89^Zr).	Specific radiotracers for environmental factors such as hypoxia ([^18^F]-FMISO), proliferation ([^18^F]-FLT) or angiogenesis.	Clinical: ~4 mm (PET) ~10mm (SPECT) Preclinical: < 1 mm (PET/SPECT)	Slow	Whole body	- Established clinical method - Non-invasive - Images biological processes and metabolic activity - Quantitative - High sensitivity (pM-nM)	- Labelling required - Low resolution - Radiation - Lacks anatomical information: combination with other modality (CT, MRI) often needed - Radiotracers can cause toxicity
	MRI	MR contrast agents as model drugs (e.g. Gd- and Mn- chelate).	- Superparamagnetic NP labelled to drugs or other NP - NP incorporating, encapsulating or labelled with MR contrast agents	Particular MRI sequences that can measure perfusion, vascular permeability, diffusion or oxygenation status.	~1 mm (clinical) ~0.1 mm (preclinical)	Slow	Whole body	- Established clinical method - Non-invasive - High spatial resolution - Physiological and anatomical information	- Contrast-agents can cause toxicity - Not compatible with certain pacemakers, metal implants, claustrophobia etc. - Indirect quantification
	CT	CT contrast agents as model drug (e.g. iodine).	Metallic NP (e.g. gold, bismuth).	Dynamic CT with contrast injection for measuring perfusion and permeability.	50-500 μm	Fast	Whole body	- Established clinical method - Non-invasive	- Radiation - Contrast-agents can cause toxicity
	US	US contrast agents as model drug (e.g. nanobubbles).	- Micro- and nanosized echogenic NP - NP labelled with US contrast agents.	- Specific ultrasound modes for measuring flow velocity and stiffness - Contrast-enhanced ultrasound to measure perfusion	50-500 μm	Fast	~ 30 cm	- Established clinical method - Non-invasive - Possible therapeutic use in sonopermeation - Anatomical and physiological information - High spatial and temporal resolution - High sensitivity (single MB detection)	- Operator dependent - Visualization difficult behind bone and air cavities
Optical Imaging	BLI/FLI	- Drug labelled with fluorescent dye - Inherently fluorescent drug - Fluorescent dyes as model drug	NP co-loaded/labelled with fluorescent dye.	- Endogenous luminescence of (tumour) cell populations (BLI) - Specific (antibody-labelled) fluorescent probes to image environmental characteristics	~5 μm	Medium	~1 cm	- Preclinically whole-body imaging possible - Combination of tracers can be used to obtain information on more than one aspect	- Surface-weighted 2D images - Labelling often required - Limited tissue depth penetration - Invasive when used clinically (biopsy or surgery needed)
	fDOT/ FMT	- Drug labelled with fluorescent dye - Fluorescent dyes as model drug	NP co-loaded/labelled with fluorescent dye.	Specific dyes to image environmental characteristics.	< 1 mm	Medium	1-2 mm	- 3D information - Possibility to combine with CT - Combination of probes can be used to obtain information on more than one aspect	- Labelling often required - Only preclinical use
	IVM	- Drug labelled with fluorescent dye - Inherently fluorescent drug - Fluorescent dyes as model drug	NP co-loaded/labelled with fluorescent dye.	Specific dyes to image environmental characteristics.	Subcellular	Fast	1-2 mm	- Preclinically non-invasive real-time method with high spatial and temporal resolution - Combination of probes can be used to obtain information on more than one aspect - High sensitivity (nM to μM)	- 2D information - Labelling often required - Only preclinical use
	Opto-acoustic	Fluorescent dye as model drug (e.g. ICG, IRDye800CW).	- Co-loading/labelling with fluorescent dye (e.g. ICG) - NP as optoacoustic contrast agent (e.g. Single-walled carbon nanotubes, gold NP)	- Endogenous contrast (e.g. Hb) - Specific (antibody-labelled) fluorescent probes to image environmental characteristics	1 μm - 1 mm	Fast	1 - 20 mm	- 3D information - Imaging at multiple scales - Penetration beyond optical diffusion limit - Combination endogenous and exogenous contrasts can be used to obtain information on more than one aspect	- Labelling often required - Operator dependent - Imaging depth is limited when the blood volume is high
Mass Spectrometry Imaging	MSI	Label-free imaging of drugs and metabolites.	Label-free imaging of NP or NP compounds (e.g. phospholipids).	-Label-free imaging endogenous compounds (e.g. metabolites, proteins, lipids). - Imaging of tumour environmental markers (e.g. hypoxia)	1 µm (IMC) 10-20 μm (MALDI) 50-200 μm (DESI) Sub-μm (SIMS)	Slow	Not applicable	- Label-free - Endogenous and exogenous compounds can be measured simultaneously to obtain information on more than one aspect - Quantitative measurement	- Invasive both preclinically and clinically (biopsy or surgery needed) - Susceptible to sampling error - Temporal information only with repeated sampling of tissue - Protocol has to be developed specifically for drug of interest

PET: positron emission tomography; SPECT: single photon emission computed tomography; NP: nanoparticle; F-MISO: fluoromisonidazole; FLT: fluorothymidine; CT: computed tomography; MRI: magnetic resonance imaging; US: ultrasound; BLI: bioluminescence imaging; FLI: fluorescence imaging; fDOT: fluorescence diffuse optical tomography; FMT: fluorescence-mediated molecular tomography; IVM: intravital microscopy; ICG: indocyanine green; Hb: haemoglobin; MSI: mass spectrometry imaging; IMC: imaging mass cytometry; MALDI: matrix assisted laser desorption ionisation; DESI: desorption electro spray ionisation; SIMS: secondary ion mass spectrometry.

## Abbreviations

ABC: accelerated blood clearance
ADC: antibody-drug conjugate
ALK5: Activin receptor-like kinase 5
BLI: bioluminescence imaging
BOLD: blood oxygenation level dependent
BPA: p-boronophenylalanine
BSH: sodium borocaptate
CEUS: contrast-enhanced ultrasound
CLSM: confocal laser scanning microscopy
CT: computed tomography
DCE: dynamic contrast enhanced
DESI: desorption electro spray ionisation
DPPG: 1,2-dipalmitoyl-sn-glycero-3-phosphoglycerol
DTPA: diethylenetriaminepentaacetic acid
DWI: diffusion weighted imaging
EGFR: epidermal growth factor receptor
EM: electron microscopy
EPR: enhanced permeability and retention
FDG: ^8^F-fluorodeoxyglucose
fDOT: fluorescence diffuse optical tomography
FES: ^18^F-fluoroestradiol
FISH: fluorescence *in situ* hybridization
FLI: fluorescence imaging
FLT: ^18^F-fluorothymidine
F-MISO: ^18^F-fluoromisonidazole
FMT-CT: fluorescence molecular tomography hybridized with computed tomography
FMT: fluorescence-mediated molecular tomography
FMX: feromoxytol
GFP: green fluorescent protein
Hb: haemoglobin
HER2: human epidermal growth factor receptor 2
HPLC: high performance liquid chromatography
ICG: indocyanine green
IFP: interstitial fluid pressure
IHC: immunohistochemistry
IVM: intravital microscopy
LA-ICP-MS: laser ablation inductively coupled plasma mass spectrometry
LC-MS: liquid chromatography mass spectrometry
IMC: imaging mass cytometry
MALDI: matrix assisted laser desorption ionisation
MPM: multiphoton microscopy
MPS: mononuclear phagocyte system
MR-HIFU: magnetic resonance guided high intensity focused ultrasound
MR-LINAC: magnetic resonance linear accelerator
MRI: magnetic resonance imaging
MSI: mass spectrometry imaging
MSOT: multispectral optoacoustic tomography
MTV: metabolic tumour volume
NIR: near-infrared
PDT: photodynamic therapy
PEG: polyethylene glycol
PEG_36-_DSPE: 1,2-distearoyl-sn-glycero-3-phosphoethanolamine conjugated with monodisperse polyethylene glycol
PET: positron emission tomography
PhCCAA: 4-Phenyl-α-cyanocinnamic acid amide
PK: pharmacokinetics
PL: photoluminescence
PLGA-PEG: poly(D,L-lactic-*co*-glycolic acid)-*b*-polyethylene glycol
PSMA: prostate specific membrane antigen
PTT: photothermal therapy
RGD: arginylglycylaspartic acid
sDHB: 2,5-dihydroxybenzoic acid
siRNA: small interfering ribonucleic acid
SIMS: secondary ion mass spectrometry
SPECT: single-photon emission computed tomography
SPION: superparamagnetic iron oxide particles
SUVmax: maximum standardized uptake values
SUVpeak: peak standardized uptake values
TAM: tumour associated macrophages
TMR: tetramethylrhodamine
TOF: time of flight
T-DM1: trastuzumab-emtansine
TLG: total lesion glycolysis
TNF: tumour necrosis factor
TOLD: tissue oxygenation level dependent
US: ultrasound
VCAM-1: vascular cell adhesion molecule 1
VEGF: vascular endothelial growth factor
